# Bioinformatic and immunological analysis reveals lack of support for measles virus related mimicry in Crohn’s disease

**DOI:** 10.1186/s12916-014-0139-9

**Published:** 2014-08-28

**Authors:** Dimitrios Polymeros, Zacharias P Tsiamoulos, Andreas L Koutsoumpas, Daniel S Smyk, Maria G Mytilinaiou, Konstantinos Triantafyllou, Dimitrios P Bogdanos, Spiros D Ladas

**Affiliations:** Hepatogastroenterology Unit, 2nd Department of Internal Medicine, National and Kapodistrian University of Athens, Attikon University General Hospital, Rimini 1, Haidari, 12462 Athens Greece; Division of Transplantation Immunology and Mucosal Biology, King’s College London School of Medicine at King’s College Hospital, London, SE5 9RS UK; Department of Rheumatology, Faculty of Medicine, School of Health Sciences, University of Thessaly, Biopolis, 41110 Larissa, Greece; 1st Department of Internal Medicine, National and Kapodistrian University of Athens, Laikon General Hospital, Athens, Greece

**Keywords:** Autoimmunity, Gastrointestinal immune response, Infectious disease, Inflammatory bowel disease

## Abstract

**Background:**

A link between measles virus and Crohn’s disease (CD) has been postulated. We assessed through bioinformatic and immunological approaches whether measles is implicated in CD induction, through molecular mimicry.

**Methods:**

The *BLAST*2p program was used to identify amino acid sequence similarities between five measles virus and 56 intestinal proteins. Antibody responses to measles/human mimics were tested by an in-house ELISA using serum samples from 50 patients with CD, 50 with ulcerative colitis (UC), and 38 matched healthy controls (HCs).

**Results:**

We identified 15 sets of significant (>70%) local amino acid homologies from two measles antigens, hemagglutinin-neuraminidase and fusion-glycoprotein, and ten human intestinal proteins. Reactivity to at least one measles 15-meric mimicking peptide was present in 27 out of 50 (54%) of patients with CD, 24 out of 50 (48%) with UC (CD versus UC, *p* = 0.68), and 13 out of 38 (34.2%) HCs (CD versus HC, *p* = 0.08). Double reactivity to at least one measles/human pair was present in four out of 50 (8%) patients with CD, three out of 50 (6%) with UC (*p* = 0.99), and in three out of 38 (7.9%) HCs (*p* >0.05 for all). Titration experiments yielded different extinction curves for anti-measles and anti-human intestinal double-reactive antibodies. Epitope prediction algorithms and three-dimensional modeling provided bioinformatic confirmation for the observed antigenicity of the main measles virus epitopic regions.

**Conclusions:**

Measles sequences mimicking intestinal proteins are frequent targets of antibody responses in patients with CD, but this reactivity lacks disease specificity and does not initiate cross-reactive responses to intestinal mimics. We conclude that there is no involvement of measles/human molecular mimicry in the etiopathogenesis of CD.

**Electronic supplementary material:**

The online version of this article (doi:10.1186/s12916-014-0139-9) contains supplementary material, which is available to authorized users.

## Background

Clinical and experimental studies have addressed the role of environmental and infectious agents in the pathogenesis of inflammatory bowel diseases (IBD) [1]. The published data are suggestive of a significant contribution of specific infectious triggers in the immune-mediated intestinal destruction seen in genetically susceptible individuals who develop Crohn’s disease (CD) [2-5]. Significant attention has been given to the role of measles virus infection and measles vaccination in the pathogenesis of the disease. This is in view of studies reporting an increasing incidence of CD in vaccinated individuals [6-9]. Early data reporting an association between measles vaccination and IBD [6,7,10] have been followed by contradictory publications that failed to identify any significant links [11]. In a similar manner, a study reporting a high incidence of CD among pregnant women affected by measles infection [7] was refuted by a subsequent report [12].

Whilst data reported in the literature have suggested that there is persistent measles infection in patients with CD [13], other laboratories were unable to note serological [14] or molecular evidence of the virus in tissues of affected individuals [15]. These inconsistencies have left unanswered the question as to whether there is a connection between measles infection (and indeed measles vaccination) and the development of CD [16,17].

In the past, we have been able to identify human proteins that are cross-recognized by microbial-specific immune responses in serum samples of patients with CD [18]. We have also been able to obtain data against a causative role of molecular mimicry between coxsackie and rubella viruses and their human mimics [19]. Vaccination providing protection against measles is achieved through a live attenuated virus vaccine contained within a triple measles, mumps, and rubella (German measles) vaccine, commonly known as the MMR. Measles vaccines develop neutralizing antibodies against measles hemagglutinin (HEMA) [20-22]. Vaccinated individuals, as well as those individuals infected by measles virus, also develop antibodies against measles virus glycoprotein (VGLF), an immunodominant viral antigen [23,24].

In the present study, we speculated that measles exposure either through vaccination or natural infection is a trigger of CD via mechanisms of molecular mimicry, involving measles and CD-related intestinal antigens.

To this end, we used protein database programs where the identification of measles/intestinal homologies was followed by construction of the peptidyl sequences spanning the homologous viral/human sequences. These mimics were then tested as antigenic targets of CD-specific humoral responses in serum samples from patients with CD or ulcerative colitis (UC) and healthy controls (HC).

## Methods

### Patients

We collected serum samples from 50 consecutive patients with CD (mean age 39.2 ± 18.2 years, range 19 to 76 years, 26 female) attending the outpatient clinic of the Hepato-Gastroenterology Unit, Attikon Hospital, University of Athens, Greece (January 2008 to December 2008). The diagnosis of CD was based on clinical, endoscopic, radiological, and histological criteria. Table 1 summarizes the demographic and clinical characteristics of the patients enrolled in the study as well as the history of exposure to measles [19]. Of these 50 patients, 39 had ileocolonic disease, ten had colonic disease, and one had isolated small-bowel disease. At the time of serum collection, 45 patients were on treatment (Table 1), and ten patients had undergone at least one surgery for CD. With regard to disease severity, 19 patients had quiescent disease, eight had mild disease, 16 had moderate disease, and seven had severe disease as classified using the Harvey-Bradshaw activity index [25].

**Table 1:** **Demographical and clinical characteristics of 50 patients with Crohn’s disease**

**Feature**	**Number**
Sex (Male/Female)	24/26
Age (mean ± SD years)	39.2 ± 18.2
Disease duration (mean ± SD years)	6.9 ± 6.1
Disease location	
Ileocolonic/Colonic/Small bowel	39/10/1
Disease behavior	
Inflammatory/Fistulizing/Stenotic	31/5/14
Disease severity	
Inactive/Mild/Moderate/Severe	19/8/16/7
Smoking (Yes/No/Unknown)	15/33/2
Surgery (Yes/No)	10/40
Medication	None: 4; 5-aminosalicylic acid: 20; azathioprine: 15; steroids:11; anti-TNFα: 11; antibiotics: 1; enteral liquid diet:1
History of measles exposure	Vaccination: 15 (30%)
	Natural infection:18 (36%)
	Unknown:^a^ 17 (34%)

^a^These patients were unable to provide information in their medical history concerning measles vaccination or exposure to measles virus infection.

Demographically matched sera from 50 patients with well-characterized UC (mean age 48.1 ± 16.1 years, range 20 to 78 years, 27 female) were included as pathologic controls. All diagnoses of UC were supported by endoscopic and histologic findings. Regarding disease extent, 12 had ulcerative proctitis, 30 had left-sided colitis, and eight had extensive disease. At the time of serum collection, five patients were on systemic steroid treatment (prednisolone) and 12 patients were on azathioprine.

We also collected serum samples from 38 healthy volunteer staff members (mean age 39.8 ± 11.4 years, range 18 to 60 years; 13 female as HC.

Experimental work complied with the principles laid down in the Declaration of Helsinki. Participating individuals gave informed consent to the work. The experimental testing of human serum samples was approved by the Attikon Hospital, National and Kapodistrian University of Athens Research Ethics Committee (registration number 9/10-10-07).

### Protein database search and analysis

The protein-protein sequence alignment BLAST2*p* program (National Center for Biotechnology Information, BLAST server available at [26], matrix: BLOSUM62) was used to align HEMA ([Swiss-Prot:P35971]; 617 amino acids) and VGLF of measles virus (MEASA [Swiss-Prot: P35973]; 550 amino acids) with human intestinal proteins, as deposited in the Swiss-Prot protein database (European Bioinformatics Institute - European Molecular Biology Laboratory, Cambridge, UK).

### Epitope prediction analysis

A hydrophobicity index (Pinsoft, Mimotopes) was provided for all the peptidyl MEASA and human intestinal (self) sequences, as described previously [18]. Further analysis of the antigenicity of peptides has been assessed using conventional computer-based tools for continuous linear B-cell epitope prediction, such as the Emini surface accessibility scale [27] and the BepiPred Linear Epitope Prediction [28] using the Immune Epitope Database and Analysis Resource platform at [29]. The Emini surface accessibility scale calculations are based on the surface accessibility scale on a product instead of an addition within the window. The accessibility profile was obtained using the formulae:

$$ Sn=\left({\displaystyle \prod_{i-1}^6\delta }n+4+i\right)\kern0.5em {(0.37)}^{-6} $$

where Sn is the surface probability, dn is the fractional surface probability value, and i varies from 1 to 6. The BepiPred prediction is based on the location of linear B-cell epitopes using a combination of a hidden Markov model and a propensity scale method [28].

### Three-dimensional modeling

Three-dimensional modeling of MEASA HEMA sequence was performed using the Cn3D visualization tool [30] and was analyzed based on the corresponding known structure of HEMA, as it has been deposited in the database [Molecular Modeling Database id:60715; PDB id: 2ZB6].

### Peptide construction

Thirty four 15-meric peptides containing measles virus and human intestinal mimics (Tables 2 and 3) were synthesized commercially and supplied at >75% purity (Mimotopes, Clayton, Victoria, Australia). All peptides were synthesized by automated solid phase fluorenylmethyloxycarbonyl chloride chemistry. A 15 amino acid-long peptidyl sequence encoding a randomly generated sequence of amino acids (-YVNQSLRPTPLEISV-) was also constructed and used as the negative control peptide, as previously described [18,19,31].

**Table 2:** **Amino acid similarities between the fusion glycoprotein of measles virus and human intestinal proteins**

	**Amino acid sequence**		**Code**	**Antigen**	**Origin**
**Set 1**	(409-506)	LIAVCLGG	VGLF MEASA	Fusion glycoprotein	Measles virus
		LIA CL G			
	(119-126)	LIATCLFG	A4P HUMAN	Intestinal membrane A4 protein	Human
**Set 2**	(350-364)	PMSPL-LQECLRGSTK	VGLF MEASA	Fusion glycoprotein	Measles virus
		P S L + ECLRG+ +			
	(187-203)	PDSALDINECLRGARR	GPC3 HUMAN	Intestinal glypican-3	Human
**Set 3**	(236-243)	YALGGDIN	VGLF MEASA	Fusion glycoprotein	Measles virus
		Y LG DIN			
	(155-162)	YILGSDIN	GPC3 HUMAN	Intestinal glypican-3	Human
**Set 4**	(44-51)	KVMTRSSH	VGLF MEASA	Fusion glycoprotein	Measles virus
		KV T+SSH			
	(382-389)	KVYTKSSH	KLF5 HUMAN	Intestinal-enriched krueppel-like factor	Human
**Set 5**	(95-102)	TQNIRPVQ	VGLF MEASA	Fusion glycoprotein	Measles virus
		+QNI+PVQ			
	(351-358)	SQNIQPVR	KLF5 HUMAN	Intestinal-enriched krueppel-like factor	Human
**Set 6**	(218-222)	GPSLR	VGLF MEASA	Fusion glycoprotein	Measles virus
		GPSLR			
	(787-791)	GPSLR	CTOG HUMAN	Colonic TOG	Human
**Set 7**	(230-235)	SIQALS	VGLF MEASA	Fusion glycoprotein	Measles virus
		SIQAL+			
	(1557-1562)	SIQALT	CTOG HUMAN	Colonic TOG	Human
**Set 8**	(507-513)	LIGIPAL	VGLF MEASA	Fusion glycoprotein	Measles virus
		LIG PAL			
	(365-371)	LIGRPAL	MGA HUMAN	Maltose-glycoamylase	Human
**Set 9**	(473-480)	AKELLESS	VGLF MEASA	Fusion glycoprotein	Measles virus
		AKE L+S+			
	(34-41)	AKESLKST	MGA HUMAN	Maltose-glycoamylase	Human
**Set 10**	(244-252)	KVLEKLGYS	VGLF MEASA	Fusion glycoprotein	Measles virus
		+VLEKL ++			
	(497-505)	QVLEKLKFT	PTK7 HUMAN	Colon carcinoma kinase-4	Human
**Set 11**	(505-514)	GGLIGIPALI	VGLF MEASA	Fusion glycoprotein	Measles virus
		G LIG+P LI			
	(252-261)	GNLIGLPLLI	CC-2 HUMAN	Colon cancer, nonpolypsis type 2	Human

**Table 3:** **Amino acid similarities between the measles hemagglutinin-neuraminidase and human intestinal proteins**

	**Amino acid sequence**		**Code**	**Antigen**	**Origin**
Set 1	(254-265)	VFEVGVIRNPGL	HEMA MEASA	Hemagglutinin-neuraminidase	Measles virus
		VFE+GV N			
	(61-72)	VFELGVTFNYNL	FAB1 HUMAN	Intestinal fatty acid-binding protein	Human
Set 2	(92-103)	LTPLFKIIGDEV	HEMA MEASA	Hemagglutinin-neuraminidase	Measles virus
		L + +IIGDE+			
	(102-113)	LNTVREIIGDEL	FAB1 HUMAN	Intestinal fatty acid-binding protein	Human
Set 3	(232-248)	YLVEKPNLSSKRSELSQ	HEMA MEASA	Hemagglutinin-neuraminidase	Measles virus
		++ + LSS+R EL Q			
	(377-393)	HVEHEETLSSRRRELIQ	GPC3 HUMAN	Intestinal glypican-3	Human
Set 4	(403-409)	KDNRIPS	HEMA MEASA	Hemagglutinin-neuraminidase	Measles virus
		K NRIPS			
	(128-134)	KLNRIPS	SUIS HUMAN	Intestinal sucrase-isomaltase	Human
Set 5	(288-293)	MVALGE	HEMA MEASA	Hemagglutinin-neuraminidase	Measles virus
		+VALGE			
	(816-821)	IVALGE	SUIS HUMAN	Intestinal sucrase-isomaltase	Human
Set 6	(584-591)	VLADSESG	HEMA MEASA	Hemagglutinin-neuraminidase	Measles virus
		V ADS SG			
	(215-222)	VCADATSG	OATP-E HUMAN	Colonic organic anion transporter	Human

Eleven sets of amino acid sequence similarities of various lengths between measles virus and virus glycoprotein fusion protein with human intestinal proteins are illustrated. The Table provides the numbering of sequence based on their local amino acid sequence homology. Fifteen-mers spanning these homologous sequences were constructed for antibody testing Amino acids appear in standard single letter code. Sequence alignment has been performed using the *BLAST2p* protein-protein comparison program. + indicates conserved or semi-conserved substitutions.

Six sets of amino acid sequence similarities between measles virus and hemagglutinin with human intestinal proteins are shown. Amino acids appear in standard single letter code. + indicates conserved or semi-conserved substitutions.

### Anti-peptide antibody reactivity by ELISA

Anti-peptide antibody reactivity was assessed by an in-house ELISA according to previously described protocols [18,32-34]. Optimal concentrations of peptides and antigens at various steps of the immunoassay were predetermined in preliminary experiments by checkerboard titration.

Serum samples from patients with CD and HCs were tested at various dilutions to determine the working dilution giving the lowest background noise and the optimal anti-peptide binding value. Also, various concentrations of individual peptides were tested (0.1, 1, 10, and 25 μg/ml) to establish the final concentration used for anti-peptide antibody binding assessment. Absorbance (optical density, OD) was read in a microplate reader (MRX; Dynex Technologies, Worthing, UK) at 490 nm. On each plate, two wells were used as blanks in which serum and peptide were omitted, and three additional wells were used for positive and negative controls. The positive control consisted of a liver kidney microsomal type 1-positive serum (titer 1/10,240) from a patient with autoimmune hepatitis type 2, which, in preliminary experiments, reacted with the immunodominant liver kidney microsomal type 1 cytochrome P4502D6 (CYP2D6)_252–271_ autoepitope. Coefficients of variation in the inter-assay and intra-assay were less than 7%. Each serum tested against experimental peptides was also tested against the control peptide. Tests based on subtracted (from the irrelevant control peptide) ODs avoid false-positive results owing to non-specific binding to unrelated peptides in hypergammaglobulinemic serum samples [18,32].

Anti-peptide antibody reactivity of the sera was considered positive when the OD of a given peptide (subtracted from that of the irrelevant control peptide) was >0.12, a cut-off higher than the mean +5 standard deviations (SD) of the absorbance values of 90 readings against the control peptide using 45 randomly selected sera (15 CD, 15 UC, and 15 HC) tested in duplicate.

Anti-measles antibodies were measured using standard ELISA (Alphadia, San Antonio, TX, USA), according to the instructions of the manufacturer.

All tests were performed using unthawed aliquots of serum samples stored at −80°C.

### Sample size calculation

We made the assumption that a significant proportion of patients with CD as well as pathological and normal controls will have single reactivities to measles peptides. However, based on our hypothesis that double reactivity to at least one homologous viral/human pair will be at least 30% more frequent in patients with CD, we calculated that a sample size of 28 subjects per group was needed to achieve power 80% with a *p* = 0.05 of statistical significance.

A sample size of 31 achieves 80% power to detect a difference of 30% in double reactivity between the null hypothesis that 25% of both the CD group and HCs show double reactivity and the alternative hypothesis that double reactivity in patients with CD is 55%, using a two-sided chi square test with continuity correction and a significance level of *p* = 0.01. Thus, a cohort of 50 patients per group could achieve statistically significant results with estimated *p* <0.01.

### Statistical analysis

Results are presented as the mean ± SD or as percentages. Comparisons between categoric values were made using the Mann-Whitney U-test, chi square test, and the Fisher exact test accordingly. Correlations between variables were assessed using the Spearman rank order correlation coefficient. A two-tailed *p-*value <0.05 was considered significant. Statistical analyses were performed using the SPSS (SPSS Inc., Chicago, IL, USA) statistical package.

## Results

### Amino acid alignment

A step-by-step approach of the design of the study related to the amino acid alignments leading to the construction of the mimicking pairs is provided as an additional file (Additional file 1).

Protein-protein BLAST2*p* analysis revealed that ten out of the 56 (18%) intestinal proteins had significant (>70%) local amino acid similarities with two of the five viral proteins, namely HEMA and/or VGLF MEASA antigens. A total of 44 pairs (involving the two viral and the ten intestinal proteins), median of four pairs (range two to seven), was further analyzed for their hydrophobicity and antigenicity indices. Amongst the 44 pairs, 17 (39%) had low hydrophobicity scores and high antigenicity scores for the viral and/or the self mimics, including 11 sets involving VGLF MEASA (Table 2) and six sets involving HEMA MEASA (Table 3). A literature search for recognition of known MEASA B-cell epitopic regions was also carried out as an additional tool to justify our decision to construct viral mimics. For each of those sets, a 15-meric peptide that encompassed the local amino acid similarity was constructed.

### Analysis of measles homologues

The alignment of VGLF and HEMA with human intestinal proteins deposited in the database led us to several observations. Amino acid similarities of VGLF with intestinal proteins were spread over the full length of the 550 amino acid-long protein, with two (amino acid 44 to 51 and 95 to 102) of them being noted in the N-terminus (amino acid 1 to 227) of the protein. Both mimics shared homologies with intestinal-enriched Krüppel-like factor-5 (see below). Of particular interest was that, in the remaining protein, there was an area defined by amino acid 218 to 252, which contained four neighboring mimics spanning amino acids 218 to 222, 230 to 235, 236 to 243, and 244 to 252. Thus, five of the 11 (45.5%) VGLF mimics were contained within a 35-long polypetidyl sequence within VGLF. The amino acid sequence 507 to 513 and its overlapping 503 to 514 also mimicked two distinct intestinal proteins (Table 2), and there was also a closely related mimic spanning amino acids 499 to 506; these three mimics defined a 16 amino acid-long sequence (amino acids 499 to 514) within VGLF. Another mimic, which was defined by amino acids 473 to 480, was also close to amino acid sequence 499 to 514.

Computer-based protein-protein comparisons of HEMA and intestinal proteins returned six hits with significant homologies. Two of those (sets 3 and 1, shown in Table 3) spanned an area of 34 amino acids (amino acids 232 to 248 and 254 to 265, respectively). These mimics were the longer of those identified, spanning 17 and 12 amino acids, respectively. The best score of identities was observed for amino acid sequence 403 to 409 (six out of seven identities, 86%).

### Analysis of human intestinal mimics

VGLF homologies of sets 2 and 3 involved intestinal glypican 3 (Table 2). The same protein also shared similarities with HEMA (set 3, Table 3). Glypican-3 [35] is a novel biomarker of malignancies of the liver and gastrointestinal system, including those of the small bowel [36,37]. Sets 4 and 5 (Table 2) included sequences originating from intestinal-enriched Krüppel-like factor 5, a zinc finger-containing transcription factor that regulates antigen-induced proliferation of intestinal epithelial cells [38]. An additional two sets of homologies between VGLF MEASA and intestinal proteins involved colonic tumor over-expressed protein (TOG), which is overexpressed in hepatic and colonic tumors [39]. Intestinal maltase glycoamylase [40] also contained two mimics of VGLF (sets 8 and 9, Table 2); this protein is mainly expressed in the small intestine, and may function as an alternative pathway for starch digestion, especially when luminal alpha-amylase activity is reduced because of immaturity or malnutrition. Its unique role in the digestion of malted dietary oligosaccharides used in food manufacturing has also been indicated [41]. Finally, colon carcinoma kinase-4 and non-polyposis type 2 (sets 10 and 11, Table 2) relate to colonic malignancies and are possibly implicated in other enteropathies.

Out of the six sets of measles HEMA/self homologies (Table 3), two involved intestinal fatty-acid-binding protein (sets 1 and 2), an early marker of intestinal destruction exclusively expressed by mature enterocytes [42,43]. This has been investigated in patients with CD, but not in great detail [44]. Also, two of the best-scored HEMA/self pairs included intestinal sucrase isomaltase (sets 4 and 5). A marked and specific decrease in sucrose isomaltase gene expression has been noted in villous enterocytes in acutely inflamed Crohn’s ileum, as compared to adjacent non-inflamed ileum and normal ileum [45]. The remaining human intestinal mimic corresponded to a colonic organic anion transporter [46], the mRNA levels of which are significantly elevated in colorectal cancers [47] and in the ileum of patients with IBD [48].

### Antibody reactivity to measles and human mimics

Reactivity to at least one measles mimic was present in 27 out of 50 patients with CD (54%; mean OD ± SD, 0.19 ± 0.11), 24 out of 50 patients with UC (48%; 0.17 ± 0.15; *p* = 0.68), and in 13 out of 38 HCs (34.2%; 0.2 ± 0.14; *p* = 0.08). Double reactivity to at least one measles/human pair was present in four out of 50 patients with CD (8%), in three out of 50 patients with UC (6%), and in three out of 38 HCs (7.9%; *p* >0.05 for all). Antibody reactivity to viral and self mimics refers to antibody binding against the 15-meric peptides constructed for immunological testing; such peptides span the sequences showing local amino acid homology as provided in Tables 2 and 3.

Significant anti-measles antibody reactivity was noted to MEASA VGLF_499–513_ (set 1, Table 2) and MEASA HEMA_234–248_ (set 3, Table 3). Overall, 24 patients with CD (48%), 22 with UC (44%), and 13 HCs (34.2%) reacted with HEMA_234–248_ (*p* >0.05 for all). Antibody reactivity to MEASA VGLF_501–515_ was present in 13 patients with CD (26%), 13 patients with UC (26%), and 10 HCs (26%). Double reactivity to MEASA HEMA_234–248_/human glypican-3_379–393_ or MEASA VGLF_499–513_/human intestinal membrane A4 was present in three patients with CD (6%), three patients with UC (6%), and three HCs (7.9%).

Using the ELISA of Alphadia, anti-measles antibodies were found in 50 patients with CD (100%), 50 with UC (100%), and 35 HCs (92%; *p* >0.05 for all).

### B-cell epitope prediction analysis

**Figure 1:**
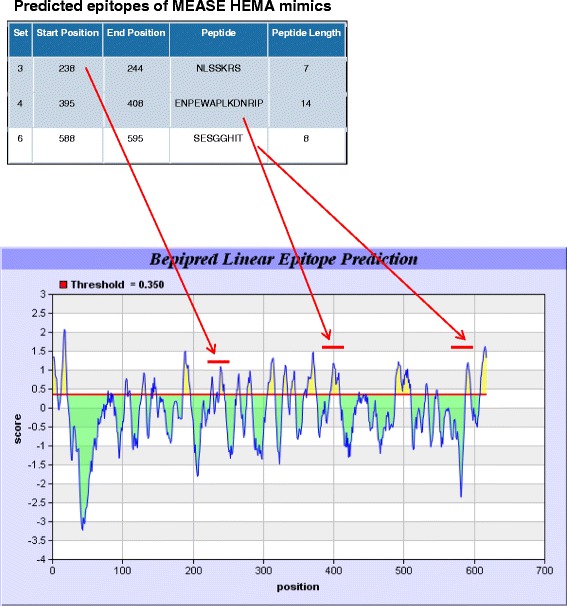
**BepiPred Linear Epitope Prediction of measles virus hemagglutinin.** The figure illustrates the exact sequences predicted to be epitopic regions that correspond to mimicking peptides (sets 3, 4, and 6 of Table 3). The exact positioning and the relevant score of all predicted epitopes is also shown.

The Emini surface accessibility scale and the BepiPred Linear Epitope Prediction tools have been used to assess the probability for antigenicity of the identified mimics. Both tools predicted the antigenicity of MEASA HEMA_234–248_ (-VEKPNLSSKRSELSQ-), giving high probability for antibody recognition of amino acids 233 to 245 (-LVEKPNLSSKRSE-) and amino acids 238 to 245 (-NLSSKRS-) (Additional file 2 and Table 3 (set 3)). The prediction tools did provide consistent results predicting the antigenicity of MEASA VGLF_499–513_ (Additional file 3). Three Emini predictions (Additional file 2; numbers 2, 4, and 12: letters in red) correspond to mimics identified through BLAST2p search (Table 2, sets 4, 5, and 9, respectively). These mimics were not predicted as potential B-cell epitopes by the BepiPred Linear Epitope Prediction program (Figure 1).

### Three-dimensional modeling

Local sequence homology modeling of MEASA HEMA illustrated the relevant exposure of the measles virus mimics in solvent-accessible surface regions of the protein (Additional file 4). As shown, MEASA HEMA_232–248_ (set 3, Table 3) is exposed to the surface of the molecule, while MEASA HEMA_254–265_ and HEMA_288–293_ are not (Additional file 4). This could explain the apparent antigenicity of MEASA HEMA_254–265._ The other two mimics do not appear to be exposed at the surface of the molecule, and this may partially explain the lack of significant antibody reactivity.

## Discussion

This is the first study to report a lack of evidence in support of a mechanism of molecular mimicry and immunological cross-reactivity involving measles virus and human intestinal antigens, potentially targeted by CD-specific immune responses. Our data clearly demonstrate that the measles vaccine *per se* is an unlikely source of an antigen-specific stimulus that could lead to the loss of immunological breakdown and the initiation of humoral immune responses targeting the intestine in patients who develop CD.

So far, studies attempting to link measles with CD have either epidemiologically associated the virus with this disease, or made efforts to provide molecular evidence of the virus through PCR and immunohistochemical analysis of tissues from affected individuals. Those studies have provided contradictory results [14-16,49-52].

Several investigators have supported the view that molecular evidence of the virus is not a prerequisite for the establishment of a link between the virus and an autoimmune disease, because measles (or other viruses) would not need to be present at the time of disease development, in a so called hit-and-run scenario [53] that involves the induction of self-destruction through a mechanisms of measles/self molecular mimicry.

At present, immunological investigation of the potential link between measles and CD has been limited to the assessment of anti-measles antibody levels in patients with CD compared to controls [14,49-51]. Equipped with sophisticated bioinformatic approaches, computer searches are now able to identify linearized molecular mimics. These data, coupled with biologically relevant sequence similarities, have provided a list of viral and human intestinal proteins worthy of investigation. However, immunological analysis of potential cross-reactive epitopes has revealed that antibody reactivity is limited to only two of 17 peptides from measles, further indicating that immunological evidence at the experimental level is required to support computerized identification of sequence fits.

The reason for the limited antigenicity probably stems from the fact that antibody responses against measles are directed against conformational epitopes, and only a small number of the identified epitopes are short and continuous in nature. Indeed, a series of published reports have provided data in support of the view that the HEMA epitopes are mostly conformational and difficult to mimic with synthetic peptides [23,24,54]. Nevertheless, those studies included data clearly demonstrating that core epitopic regions within the large epitopes of HEMA and VGLF are identical to, or largely overlap with, those identified in the current study.

Although these two mimics (one from HEMA and one from VGLF) are recognized by more than half of the tested serum samples, this reactivity is comparable amongst patients with CD and UC and HCs. This can be easily explained given that previously reported B-cell epitope mapping studies have also reported these to be dominant epitopes [23,24,54,55]. However, the intestinal mimics of the measles epitopes are virtually unreactive.

Though our sequence alignment provided a series of viral-mimicking intestinal antigens, none of those appear to be close enough to their human mimics to share immunogenic determinants. Thus, neither the VGLF_499–513_ mimic from human intestinal membrane A4_119–133_, nor that of glypican-3_379–393_ mimicking HEMA_234–248_ are cross-recognized. This is not the first time that glypican-3 sequences have been found to share significant homologies with CD-related microbial sequences. In fact, seven out of 16 mimicking sets between *Mycobacterium avium paratuberculosis* and human intestinal proteins involved sucrose isomaltase and glypican 3, but these homologs were not recognized by antibodies in sera from patients with CD [18]. The wealth of data provided so far is largely supportive of a role of glypican-3 as a marker of hepatocellular rather than gastrointestinal malignancy [37,56], further indicating the unlikely possibility of this protein serving as a cryptic intestinal-specific autoantigen in IBD.

Sucrose isomaltase and colonic TOG sequences are homologous to MEASA HEMA and VGLF, but are not recognized by CD-specific antibodies. This finding is of interest given that our previous findings, which indicate peptide-specific antibody reactivity in a TOG, mimic *Mycobacterium avium paratuberculosis* in 50% of patients with CD [18].

Two further points need to be made. Despite HEMA being longer than VGLF (617 versus 550 amino acids), more significant similarities have been identified with the latter than the former. We considered this an intriguing finding and we further explored the antigenic potential of the two proteins using a combination of epitope prediction algorithms, such as Emini and BepiPred prediction algorithms for linear epitopes (Figure 1 and Additional files 3 and 2). These tools provided theoretical support for the antigenic potential of most of the HEMA and VGLF mimicking sequences that we constructed, the best hits for HEMA being amongst those that were identified as antibody targets. Also, three-dimensional analysis of the known structure of HEMA has led us to understand why there are peptides that react like HEMA_232–248_ and others that are not recognized, providing bioinformatic evidence of the observed antigenicity (Additional file 4).

## Conclusion

Our study provides new evidence against a potential link between measles and CD, based on the lack of B-cell cross-reactivity amongst viral and intestinal mimics. If these findings are confirmed in larger independent studies, they may shed some light on the provocative view of an apparent immunopathogenetic connection between measles virus and CD.

### Conference presentation

Preliminary data of this work was presented at the Gastro 2009 UEGW/WCOG conference, London, November 2009, as a poster entitled “Molecular mimicry and immunological cross-reactivity between measles virus and human intestinal proteins is not a feature of Crohn’s disease.” The abstract of this presentation was published in *Gut* 2009; 58(Suppl II):A154.

## Additional files

Additional file 1:
**Step-by-step approach of the design of the study.**
Additional file 2:
**Emini Surface Accessibility Prediction of measles virus hemagglutinin.** The figure illustrates the exact sequences predicted to be epitopic regions which correspond to mimicking peptides (sets 3 and 4 of Table 3) (seen in red letters within the amino acid protein sequence), as well as the position and the scores of the predicted epitopes within the protein. Potential epitopic region areas are illustrated within yellow wavelengths. Note that several sequences are predicted to be epitopic, but only two of those correspond to measles virus hemagglutinin mimics (indicated with red arrows). Additional file 3:
**(a) Emini Surface Accessibility Prediction of measles virus glycoprotein fusion and (b) BepiPred Linear Epitope Prediction of measles virus fusion glycoprotein.** Note that the three Emini predictions (numbers 2, 4, and 12: letters in red) correspond to mimics identified through BLAST2p search (Table 2, sets 4, 5, and 9, respectively). However, the corresponding mimics are not predicted as potential B-cell epitopes by the BepiPred Linear Epitope Prediction program. Additional file 4:
**Three-dimensional prediction model of the amino acids from the dimeric hemagglutinin of measles virus (PDB Accession number: 2ZB6) is presented in the form of a wire worm backbone (green/purple).** A space fill (green/purple) illustration is used to show three measles mimics (in yellow, indicated by arrows) corresponding to the viral sequences given in sets 1, 3, and 5. Helix and strand elements are also shown (low structure). The structure was analyzed with the Cn3D visualization tool.

## Abbreviations

BLAST2*p*: Basic local alignment search tool
CD: Crohn’s disease
ELISA: enzyme-linked immunosorbent assay
HC: healthy control
HEMA: hemagglutinin
IBD: inflammatory bowel disease
MEASA: Measles virus (strain Edmonston-AIK-C vaccine)
OD: optical density
PCR: polymerase chain reaction
SD: standard deviation
TNFα: tumor necrosis factor alpha
UC: ulcerative colitis
VGLF: virus glycoprotein fusion
