# Supplementary material for: Bioinformatic and immunological analysis reveals lack of support for measles virus related mimicry in Crohn’s disease
Source: BMC Med. 2014 Aug 28;12:139. doi: 10.1186/s12916-014-0139-9 (PMC4171545; doi:10.1186/s12916-014-0139-9)
Supplement: Additional file 1: — Step-by-step approach of the design of the study. [file 12916_2014_139_MOESM1_ESM.ppt]

## Slide 1
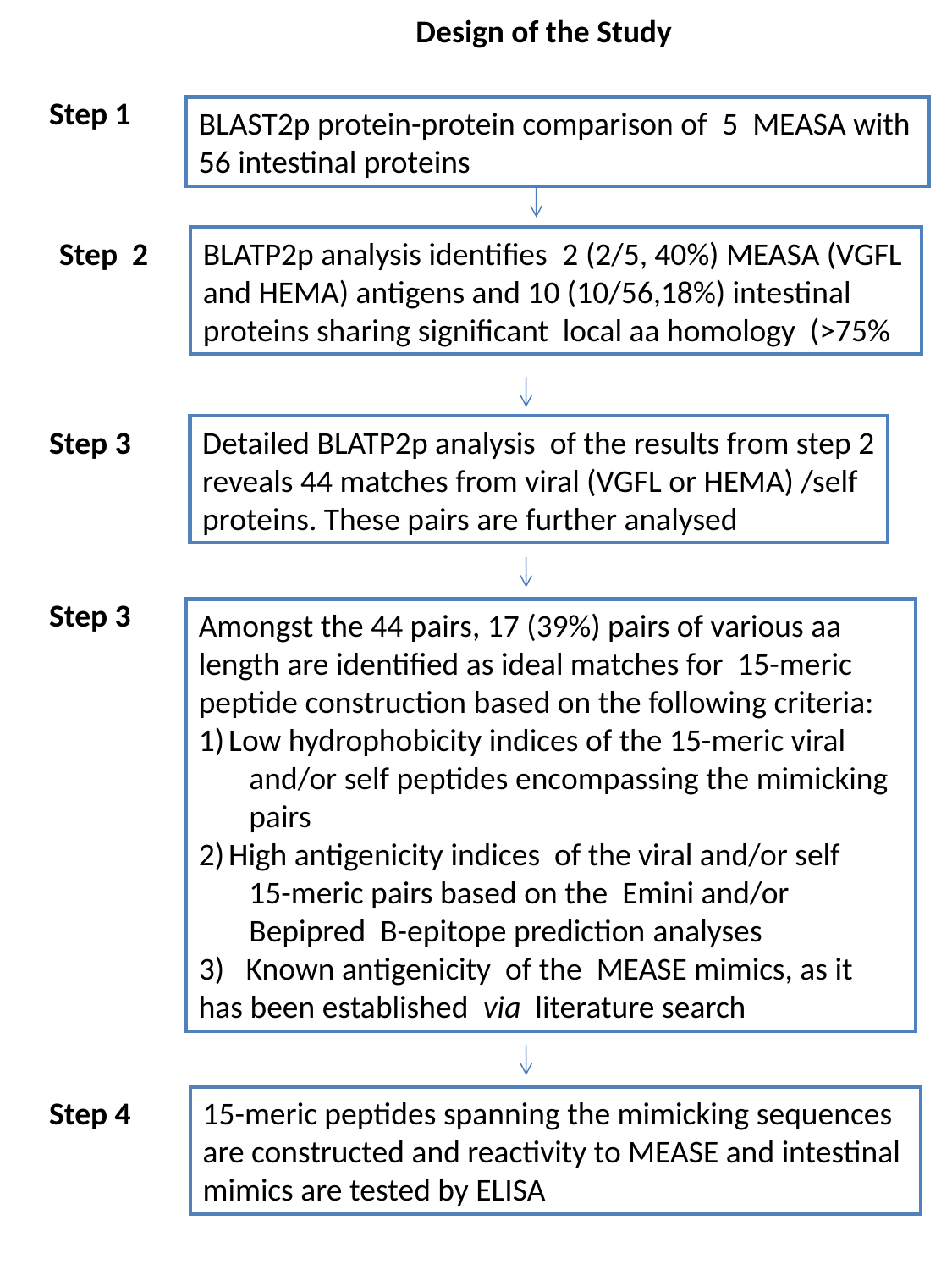

Design of the Study
Step 1
BLAST2p protein-protein comparison of 5 MEASA with 56 intestinal proteins
Step 2
BLATP2p analysis identifies 2 (2/5, 40%) MEASA (VGFL
and HEMA) antigens and 10 (10/56,18%) intestinal
proteins sharing significant local aa homology (>75%
Step 3
Detailed BLATP2p analysis of the results from step 2
reveals 44 matches from viral (VGFL or HEMA) /self
proteins. These pairs are further analysed
Step 3
Amongst the 44 pairs, 17 (39%) pairs of various aa length are identified as ideal matches for 15-meric peptide construction based on the following criteria:
Low hydrophobicity indices of the 15-meric viral
 and/or self peptides encompassing the mimicking
 pairs
High antigenicity indices of the viral and/or self
 15-meric pairs based on the Emini and/or
 Bepipred B-epitope prediction analyses
3) Known antigenicity of the MEASE mimics, as it has been established via literature search
Step 4
15-meric peptides spanning the mimicking sequences
are constructed and reactivity to MEASE and intestinal
mimics are tested by ELISA
