# Supplementary figures and images for: Bioinformatic and immunological analysis reveals lack of support for measles virus related mimicry in Crohn’s disease
Source: BMC Med. 2014 Aug 28;12:139. doi: 10.1186/s12916-014-0139-9 (PMC4171545; doi:10.1186/s12916-014-0139-9)

## Slide 1
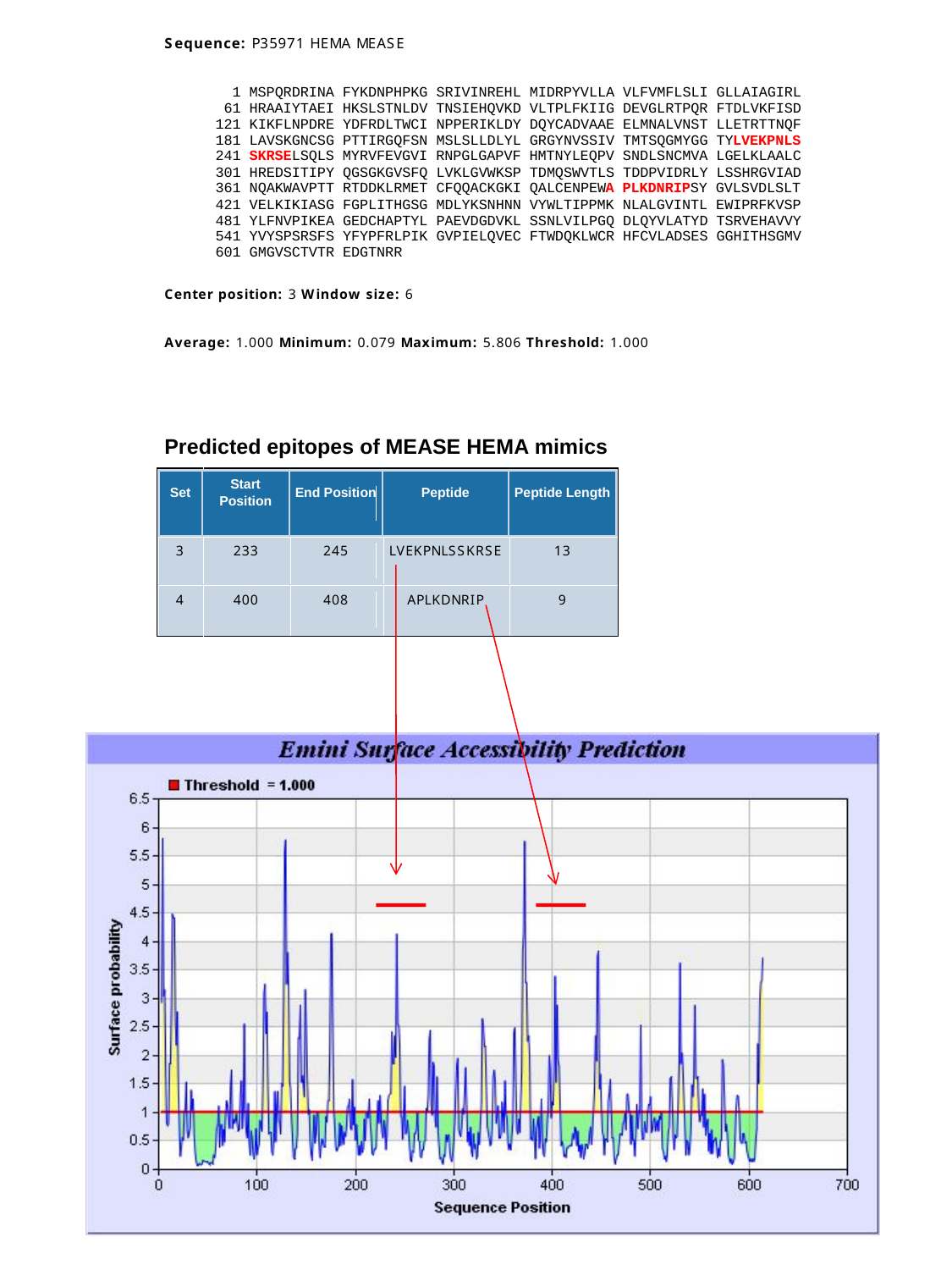

Supplement: Additional file 2: — Emini Surface Accessibility Prediction of measles virus hemagglutinin. The figure illustrates the exact sequences predicted to be epitopic regions which correspond to mimicking peptides (sets 3 and 4 of Table 3) (seen in red letters within the amino acid protein sequence), as well as the position and the scores of the predicted epitopes within the protein. Potential epitopic region areas are illustrated within yellow wavelengths. Note that several sequences are predicted to be epitopic, but only two of those correspond to measles virus hemagglutinin mimics (indicated with red arrows). [file 12916_2014_139_MOESM2_ESM.ppt]

## Slide 1
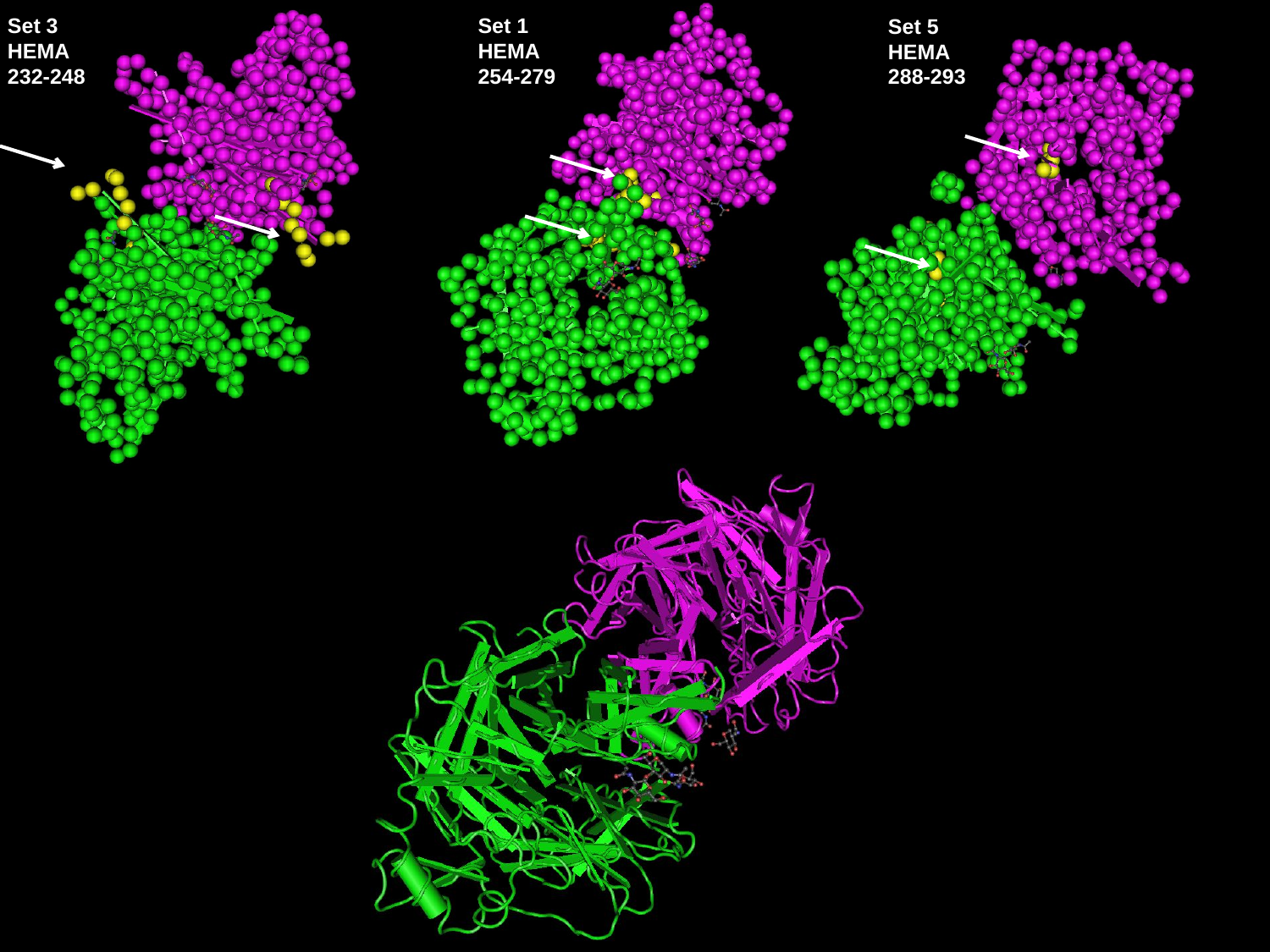

Set 3
HEMA
232-248
Set 1
HEMA
254-279
Set 5
HEMA
288-293

Supplement: Additional file 4: — Three-dimensional prediction model of the amino acids from the dimeric hemagglutinin of measles virus (PDB Accession number: 2ZB6) is presented in the form of a wire worm backbone (green/purple). A space fill (green/purple) illustration is used to show three measles mimics (in yellow, indicated by arrows) corresponding to the viral sequences given in sets 1, 3, and 5. Helix and strand elements are also shown (low structure). The structure was analyzed with the Cn3D visualization tool. [file 12916_2014_139_MOESM4_ESM.ppt]
