# Supplementary material for: Bioinformatic and immunological analysis reveals lack of support for measles virus related mimicry in Crohn’s disease
Source: BMC Med. 2014 Aug 28;12:139. doi: 10.1186/s12916-014-0139-9 (PMC4171545; doi:10.1186/s12916-014-0139-9)
Supplement: Additional file 3: — (a) Emini Surface Accessibility Prediction of measles virus glycoprotein fusion and (b) BepiPred Linear Epitope Prediction of measles virus fusion glycoprotein. Note that the three Emini predictions (numbers 2, 4, and 12: letters in red) correspond to mimics identified through BLAST2p search (Table 2, sets 4, 5, and 9, respectively). However, the corresponding mimics are not predicted as potential B-cell epitopes by the BepiPred Linear Epitope Prediction program. [file 12916_2014_139_MOESM3_ESM.doc]

## Suppl Figure 2

## Emini Surface Accessibility Prediction of measles virus fusion glycoprotein.

**Sequence:** p35973

        1 MGLKVNVSAI FMAVLLTLQT PTGQIHWGNL SKIGVVGIGS ASYKVMTRSS HQSLVIKLMP 
       61 NITLLNNCTR VEIAEYRRLL RTVLEPIRDA LNAMTQNIRP VQSVASSRRH KRFAGVVLAG 
      121 AALGVATAAQ ITAGIALHQS MLNSQAIDNL RASLETTNQA IEAIRQAGQE MILAVQGVQD 
      181 YINNELIPSM NQLSCDLIGQ KLGLKLLRYY TEILSLFGPS LRDPISAEIS IQALSYALGG 
      241 DINKVLEKLG YSGGDLLGIL ESRGIKARIT HVDTESYLIV LSIAYPTLSE IKGVIVHRLE 
      301 GVSYNIGSQE WYTTVPKYVA TQGYLISNFD ESSCTFMPEG TVCSQNALYP MSPLLQECLR 
      361 GSTKSCARTL VSGSFGNRFI LSQGNLIANC ASILCKCYTT GTIINQDPDK ILTYIAADHC 
      421 PVVEVNGVTI QVGSRRYPDA VYLHRIDLGP PILLERLDVG TNLGNAIAKL EDAKELLESS 
      481 DQILRSMKGL SSTCIVYILI AVCLGGLIGI PALICCCRGR CNKKGEQVGM SRPGLKPDLT 
      541 GTSKSYVRSL

**Center position:** 3   **Window size:** 6


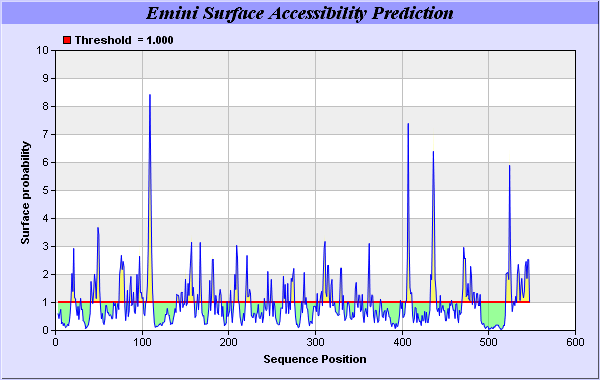


**Average:** 1.000   **Minimum:** 0.028   **Maximum:** 8.441   **Threshold:** 1.000

*Bepipred Linear Epitope Prediction Bepipred Linear Epitope Prediction* Bottom of Form

#### Predicted epitopes of MEASE VGLF mimics

| **Set** | **Start Position** | **End Position** | **Peptide** | **Peptide Length** |
| --- | --- | --- | --- | --- |
| 1 | 18 | 23 | LQTPTG | 6 |
| 2 | 44 | 51 | KVMTRSSH | 8 |
| 3 | 74 | 79 | AEYRRL | 6 |
| 4 | 96 | 101 | QNIRPV | 6 |
| 5 | 105 | 112 | ASSRRHKR | 8 |
| 6 | 153 | 160 | SLETTNQA | 8 |
| 7 | 206 | 211 | LLRYYT | 6 |
| 8 | 220 | 225 | SLRDPI | 6 |
| 9 | 309 | 316 | QEWYTTVP | 8 |
| 10 | 404 | 411 | INQDPDKI | 8 |
| 11 | 433 | 439 | GSRRYPD | 7 |
| 12 | 469 | 480 | KLEDAKELLESS | 12 |
| 13 | 520 | 526 | RCNKKGE | 7 |

## Bepipred Linear Epitope Prediction of measles virus fusion glycoprotein.

Top of Form


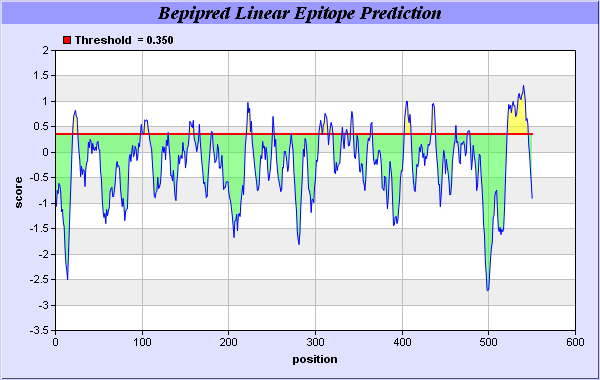


**Average:**-0.251   **Minimum:**-2.721   **Maximum:**1.315   **Threshold:**

#### Predicted epitopes:

| **No.** | **Start Position** | **End Position** | **Peptide** | **Peptide Length** |
| --- | --- | --- | --- | --- |
| 1 | 20 | 25 | TPTGQI | 6 |
| 2 | 98 | 108 | IRPVQSVASSR | 11 |
| 3 | 130 | 130 | Q | 1 |
| 4 | 155 | 159 | ETTNQ | 5 |
| 5 | 166 | 166 | Q | 1 |
| 6 | 180 | 181 | DY | 2 |
| 7 | 221 | 226 | LRDPIS | 6 |
| 8 | 251 | 252 | YS | 2 |
| 9 | 272 | 272 | V | 1 |
| 10 | 304 | 307 | YNIG | 4 |
| 11 | 309 | 314 | QEWYTT | 6 |
| 12 | 317 | 320 | KYVA | 4 |
| 13 | 331 | 331 | E | 1 |
| 14 | 335 | 337 | TFM | 3 |
| 15 | 341 | 343 | TVC | 3 |
| 16 | 364 | 365 | KS | 2 |
| 17 | 403 | 410 | IINQDPDK | 8 |
| 18 | 434 | 438 | SRRYP | 5 |
| 19 | 462 | 462 | N | 1 |
| 20 | 476 | 478 | LLE | 3 |
| 21 | 522 | 545 | NKKGEQVGMSRPGLKPDLTGTSKS | 24 |
